# Intracellular cAMP signaling-induced Ca^2+^ influx mediated by calcium homeostasis modulator 1 (CALHM1) in human odontoblasts

**DOI:** 10.1007/s00424-024-03038-4

**Published:** 2024-11-12

**Authors:** Maki Kimura, Sachie Nomura, Takehito Ouchi, Ryuya Kurashima, Rei Nakano, Hinako Sekiya, Hidetaka Kuroda, Kyosuke Kono, Yoshiyuki Shibukawa

**Affiliations:** 1 Department of Physiology, Tokyo Dental College, Tokyo, 101-0061 Japan; 2 Laboratory for Mucosal Immunity, Center for Integrative Medical Sciences, RIKEN Yokohama Institute, Yokohama, 230-0045 Japan; 3 Japan Animal Specialty Medical Institute (JASMINE), Yokohama, 224-0001 Japan; 4 Department of Endodontics, Tokyo Dental College, Tokyo, 101-0061 Japan; 5 Department of Dental Anesthesiology, Kanagawa Dental University, Yokosuka, 238-8570 Japan

**Keywords:** Odontoblasts, Cyclic AMP, Ca^2+^ signaling, Adenylyl cyclase, Protein kinase A, Calcium homeostasis modulator 1

## Abstract

In odontoblasts, intracellular Ca^2+^ signaling plays key roles in reactionary dentin formation and generation of dentinal pain. Odontoblasts also express several G_s_ protein-coupled receptors that promote production of cyclic AMP (cAMP). However, the crosstalk between intracellular cAMP and Ca^2+^ signaling, as well as the role of cAMP in the cellular functions of odontoblasts, remains unclear. In this study, we measured intracellular cAMP levels and intracellular free Ca^2+^ concentration ([Ca^2+^]_i_). We also investigated the effect of intracellular cAMP on mineralization by the odontoblasts. In the presence of extracellular Ca^2+^, the application of forskolin (adenylyl cyclase activator) or isoproterenol (G_s_ protein-coupled beta-2 adrenergic receptor agonist) increased intracellular cAMP levels and [Ca^2+^]_i_ in odontoblasts. The [Ca^2+^]_i_ increases could not be observed by removing extracellular Ca^2+^, indicating that cAMP is capable to activate Ca^2+^ entry. Forskolin-induced [Ca^2+^]_i_ increase was inhibited by a protein kinase A inhibitor in odontoblasts. The [Ca^2+^]_i_ increase was sensitive to Gd^3+^, 2APB, or Zn^2+^ but not verapamil, ML218, or La^3+^. In immunofluorescence analyses, odontoblasts were immunopositive for calcium homeostasis modulator 1 (CALHM1), which was found close to ionotropic ATP receptor subtype, P2X_3_ receptors. When CALHM1 was knocked down, forskolin-induced [Ca^2+^]_i_ increase was suppressed. Alizarin red and von Kossa staining showed that forskolin decreased mineralization. These findings suggest that activation of adenylyl cyclase elicited increases in the intracellular cAMP level and Ca^2+^ influx via protein kinase A activation in odontoblasts. Subsequent cAMP-dependent Ca^2+^ influx was mediated by CALHM1 in odontoblasts. In addition, the intracellular cAMP signaling pathway in odontoblasts negatively mediated dentinogenesis.

## Introduction

Odontoblasts act as dentin-forming cells in physiological and pathological settings, as well as mechanosensory receptor cells in generating dentinal pain, such as that during dentin hypersensitivity, following a variety of stimuli on the dentin surface. Stimulation of the dentin surface induces membrane stretch evoked by dentinal fluid movement, resulting in an increase in intracellular Ca^2+^ concentration via Ca^2+^ influx through mechanosensitive ion channels, Piezo1, transient receptor potential vanilloid subfamily member 1 (TRPV1), TRPV2, TRPV4, and TRP ankylin 1 (TRPA1) channels in odontoblasts [32, 36, 37, 42, 43, 45, 54, 55]. An increase in the intracellular Ca^2+^ concentration stimulates the release of ATP from pannexin-1 channels (PANX-1) and glutamate from glutamate-permeable anion channels in the odontoblasts, which act as intercellular transmitters from odontoblasts to the neurons [36, 37, 45]. The released ATP binds to ionotropic ATP receptor subtype, P2X_3_ (P2X_3_) receptors, and glutamate activates metabotropic glutamate receptors in the trigeminal ganglion (TG) neurons that innervate the dental pulp. Activation of the P2X_3_ or metabotropic glutamate receptors mediates the generation of action potentials in TG neurons, causing dentinal pain [37, 43, 45]. Meanwhile, the intracellular Ca^2+^, whose levels are increased via Ca^2+^ influx through mechanosensitive ion channels, is extruded by Na^+^-Ca^2+^ exchangers (NCX) [44, 53] and plasma membrane Ca^2+^-ATPase (PMCA) [18] to the extracellular medium, resulting in reactionary dentin formation and mineralization. Therefore, the intracellular Ca^2+^ signaling in odontoblasts plays a critical role in dentin formation and dentinal pain generation.

G protein-coupled receptors (GPCRs) are seven transmembrane-spanning proteins regulated by G proteins [50]. The G protein is a heterotrimer composed of α, β, and γ subunits and classified into four main families (G_s_, G_i_, G_q_, and G_12/13_) depending on the sequence and functional similarities of Gα subunits [6]. The G_s_ and G_i_ proteins regulate adenylyl cyclase activity. The activation of G_s_ protein-coupled receptors stimulates adenylyl cyclase and facilitates the production of cyclic AMP (cAMP). The increase in intracellular cAMP activates protein kinase A (PKA) [28, 50]. The G_s_ protein-coupled receptors are expressed in various tissues and control many physiological functions, such as vision, olfactory perception, synthesis and secretion of hormones, and cardiac activation [50]. Odontoblasts express several G_s_ protein-coupled receptors, including calcitonin gene-related peptide (CGRP), prostaglandin I_2_, adenosine A_2A_, dopamine D_1_, and 5-hydroxytriptamine 4 receptors [22, 41]. Beta-2 adrenergic (β_2_) receptors, which are representative G_s_ protein-coupled receptors, are also expressed in odontoblasts [10]. However, the details of the intracellular cAMP signaling pathway, the role of cAMP in cellular function, and the participation of cAMP in Ca^2+^ signaling in odontoblasts remain unclear. In the present study, we examined intracellular cAMP and Ca^2+^ signaling induced by the activation of adenylyl cyclase and β_2_ receptors and the crosstalk between these signaling in human odontoblasts. In addition, we investigated the effect of intracellular cAMP on mineralization by human odontoblasts.

## Materials and methods

### Human odontoblast cell culture

The human odontoblast cell line (HOB cell) was obtained from a healthy third molar and immortalized cells via the human telomerase transcriptase gene transfection [21]. The HOB cells were provided by Dr Masae Kitagawa of Hiroshima University, Japan, and Dr Takashi Muramatsu of Tokyo Dental College, Japan. HOB cells express mRNA corresponding to dentin sialophosphoprotein (DSPP), type I collagen, alkaline phosphatase, and bone sialoprotein [21]. These cells were used for experiments until approximately 40 passages, and we confirmed these cells were immunoreactive for dentin matrix protein-1, DSPP, and nestin, which are odontoblast markers [32]. Therefore, this cell line possesses odontoblastic properties. We cultured HOB cells in basal medium (α-minimum essential medium; Thermo Fisher Scientific Inc., Waltham, MA, USA) supplemented with 10% fetal bovine serum, 1% penicillin/streptomycin (Thermo Fisher Scientific Inc.), and 1% amphotericin B (Sigma Ardrich, St. Louis, MO, USA) at 37 °C in an atmosphere containing 5% CO_2_. HOB cells were used for the determination of intracellular cAMP levels and [Ca^2+^]_i_, immunofluorescence, and mineralization assays.

### Ethical approval

We treated all animals in accordance with the guiding principles for the care and use of animals in the field of physiologic sciences approved by the Council of the Physiological Society of Japan and the American Physiological Society. We performed all animal experiments following the guidelines established by the U.S. National Institutes of Health regarding the care and use of animals for experimental procedures, as well as the UK Animals (Scientific Procedures) Act of 1986. The present study was approved by the Ethics Committee of our institute (No. 220301, 230301 and 240301).

### Fluorescence measurement of intracellular cAMP levels

A live-cell cAMP assay was performed using the green cAMP BacMam sensor. The sensor was a vector containing a gene encoding a fluorescent protein and a cAMP-sensitive fluorescent biosensor (green upward cAMP difference detector in situ; Montana Molecular, Bozeman, MT, USA). HOB cells were seeded at a density of 20,000–25,000 cells/mL in 35 mm dish (Nippon Genetics, Tokyo, Japan) and incubated at 37 °C in an atmosphere containing 5% CO_2_ for 72 h. The medium was then replaced with fresh basal medium containing 16.7% cAMP BacMam sensor and 0.4% Na-butyrate, and the cAMP sensor was transfected into HOB cells by incubation at 37 °C for 36 h. The cAMP sensor-transfected HOB cells were washed with a fresh standard extracellular solution. A dish containing cAMP sensor-transfected HOB cells was mounted on the stage of a microscope (IX73, Evident Co., Tokyo, Japan) and examined using HCImage software (Hamamatsu Photonics, Shizuoka, Japan). The microscope was equipped with an excitation wavelength selector and intensified charge-coupled device camera system (Hamamatsu Photonics). The intracellular cAMP level was measured at an emission wavelength of 517 nm and excitation wavelength of 506 nm (F_cAMP_), and was identified based on the fluorescence ratio (F/F_0 cAMP_ unit); the fluorescence value (F_cAMP_) was normalized to the resting value (F_0 cAMP_). To obtain the peak F/F_0 cAMP_ value, the base value of F/F_0 cAMP_ was normalized by the mean value for 30 s just before each application of pharmacological agents. The F/F_0 cAMP_ base value was set to 1.0. We applied a standard extracellular solution containing an activator or inhibitor of adenylyl cyclase or a β_2_ receptor agonist using a perfusion system (ValueLink8.2 Controller; AutoMate Scientific, Berkeley, CA, USA). All experiments were performed at 28 ± 2 °C.

### Fluorescence measurement of [Ca^2+^]_i_

[Ca^2+^]_i_ was measured by fluorescence of fura-2-acetoxymethyl ester (Dojindo Laboratories, Kumamoto, Japan). The cAMP sensor-transfected or non-transfected HOB cells were loaded with 10 µM fura-2-acetoxymethyl ester and 0.1% (w/v) pluronic acid F-127 (Thermo Fisher Scientific Inc.) in standard extracellular solution, followed by incubation at 37 °C for 60 min in an atmosphere containing 5% CO_2_. We then rinsed the fura-2-loaded HOB cells with a fresh standard extracellular solution. A dish containing fura-2-loaded HOB-transfected or non-transfected with the cAMP sensor was placed on the stage of the microscope (IX73 or IX71; Evident Co.). We performed fura-2 fluorescence measurements with an emission wavelength of 510 nm by excitation wavelength of 340 (F340) and 380 nm (F380); [Ca^2+^]_i_ represented the fluorescence intensity ratio of F340 to F380 (R_F340/F380_). The R_F340/F380_ value (F_Ca_) was normalized to the resting value (F_0 Ca_) and denoted in F/F_0 Ca_ units. To obtain the peak F/F_0 Ca_ value, the F/F_0 Ca_ base value was normalized by the mean value for 30 s just before each application of pharmacological agents. The F/F_0 Ca_ base value was set to 1.0. We applied a standard extracellular solution with an adenylyl cyclase activator, a PKA inhibitor, or each channel antagonist using the perfusion system (ValueLink8.2). All experiments were performed at 28 ± 2 °C.

### Immunostaining analysis

We cultured HOB cells in 8-well glass chambers (AGC Techno Glass Co., Ltd., Shizuoka, Japan) for 1 day. After fixation with 4% paraformaldehyde (FUJIFILM Wako Pure Chemical Co., Osaka, Japan), the cells were rinsed with 1 × PBS (Thermo Fisher Scientific Inc.). After incubation with blocking buffer (Nacalai Tesque, Kyoto, Japan) containing 0.3% Triton-X100 for 60 min at room temperature, we applied rabbit polyclonal anti-calcium homeostasis modulator 1 (CALHM1) (Proteintech Group Inc., Rosemont, IL, USA; 22042–1-AP, 1:200) and mouse monoclonal anti-Tom20 (Santa Cruz Biotechnology, Dallas, TX, USA; sc-17764, F-10, 1:200) to detect expression of human mitochondria for 6 h at room temperature. We then applied secondary antibodies (Alexa Fluor® 488 donkey anti-rabbit and Alexa Fluor® 568 donkey anti-mouse; Thermo Fisher Scientific Inc.) for 60 min at room temperature. The stained samples were placed in a mounting medium containing 4,6-diamidino-2-phenylindole (Abcam, Cambridge, UK). The samples were analyzed using a fluorescence microscope (BZ9000; KEYENCE Co., Osaka, Japan). Cells that were incubated in the first incubation fluid without the primary antibody were used as a negative control (data not shown).

To examine the localization pattern of CALHM1 in odontoblasts and P2X_3_ receptors, mandibles were dissected from 19-week-old mice under 3% isoflurane anesthesia. Dissected mandibles were dipped in 4% paraformaldehyde for 24 h at 4 °C. After decalcification with 10% ethylenediaminetetraacetic acid (MUTO Pure Chemicals Co., LTD, Tokyo, Japan) for 4 weeks at 4 °C, samples of the mandibles were immersed in 10%, 20%, and 30% sucrose solution, and encapsulated in O.C.T. (optimal cutting temperature) compound (Sakura Finetek Japan., Tokyo, Japan; 4583). The frozen tissue sections with 10 µm thickness were prepared using a Leica CM1950 cryostat (Leica Biosystems, Wetzlar, Germany). After fixation with 4% paraformaldehyde, the sections were rinsed with 1 × PBS. After incubation with blocking buffer (Nacalai Tesque) containing 0.3% Triton-X100 for 60 min at room temperature, we applied rabbit polyclonal anti-CALHM1 (1:200) and mouse monoclonal anti-P2X3 (Santa Cruz Biotechnology, sc-390572, B-5, 1:200) overnight at 4 °C. We applied then secondary antibodies (Alexa Fluor® 488 goat anti-rabbit and Alexa Fluor® 555 goat anti-mouse; Thermo Fisher Scientific Inc.) for 60 min at room temperature. The stained samples were placed in a mounting medium containing 4,6-diamidino-2-phenylindole (Abcam). The samples were analyzed using a microscope (Axioobserver7; Carl Zeiss Co., Ltd. Oberkochen, Germany). The microscope was equipped with a confocal microscope (LSM900; Carl Zeiss). Sections incubated in the first incubation fluid without the primary antibody were used as a negative control (data not shown).

### Knocked-down CALHM1 expression by gene silencing with short hairpin RNA

To produce the HOB cells transfected by short hairpin RNA (shRNA), including a vector specific for human CALHM1 (shRNA-CALHM1 transfected cells) or an empty vector control (shRNA-control transfected cells), we used the Lenti-X™ 293T cell line (Takara Bio Inc., Shiga, Japan). Packaging plasmids pCAG-HIVgp (RIKEN BioResource Research Center, Tsukuba, Japan, RDB04394) and pCMV-VSV-G-RSV-Rev (RIKEN BioResource Research Center, RDB04393) as well as lentiviral vectors were transfected into the Lenti-X™ 293T cells. We used lentiviral vectors specifically targeting human CALHM1 (Sigma-Aldrich) and a TRC2 empty vector control (Sigma-Aldrich) in this experiment. The supernatant of the Lenti-X™ 293T cells including the lentivirus was harvested after 72 h and used to infect HOB cells. HOB cells with 60–80% confluency were transfected with lentiviral vector particles containing shRNA and incubated for 72 h. We cultured the transfected HOB cells for 72 h after screening with 1 mg/mL puromycin.

### Mineralization assay

HOB cells were cultured to full confluency in basal medium containing 1.8 mM Ca^2+^, which was then changed to a mineralization medium (prepared by adding 50 µg/mL ascorbic acid and 10 mM β-glycerophosphate to the basal medium) at 37 °C in 5% CO_2_. To demonstrate the effects of increased intracellular cAMP on mineralization by HOB cells, the cells were cultured in a mineralization medium without (as a control) or with an adenylyl cyclase activator, forskolin (FSK), for 21 days. The mineralization medium with or without FSK was changed twice a week during the 21-day culture period. Calcium deposition was detected using alizarin red staining, and calcium phosphate deposition was assessed using von Kossa staining [18, 20, 23]. Mineralizing potency was analyzed using ImageJ software (NIH, Bethesda, Maryland, USA). Images obtained using a digital camera (Sony, Tokyo, Japan) were converted to 8-bit and reversed grayscale images. The regions of interest (ROIs) in each well were determined to measure the mean luminance intensities of the total pixel numbers (I) in the ROI. The intensities (I) were normalized to the mean values of the intensities in the areas without cells (I_0_). Mineralization potency was shown as I/I_0_ units for alizarin red and von Kossa staining.

### Solution and reagents

The standard extracellular solution was Krebs solution containing: 136 mM NaCl, 5 mM KCl, 2.5 (or 0) mM CaCl_2_, 0.5 mM MgCl_2_, 10 mM HEPES, 10 mM glucose, and 12 mM NaHCO_3_ (pH 7.4, Tris). FSK, KT5720, GdCl_3_, 2APB, ML218, and verapamil (VRP) were obtained from TOCRIS Biosciences (Bristol, UK). LaCl_3_ was purchased from Sigma Aldrich. All other reagents were purchased from FUJIFILM Wako Pure Chemical Co. Stock solutions were prepared in dimethyl sulfoxide for FSK, KT5720, 2APB, ML218, and VRP. The stock solutions for LaCl_3_, ZnSO_4_, and GdCl_3_ were prepared in MilliQ water. Stock solutions were diluted with standard extracellular solutions to the appropriate concentrations before use.

### Statistical and offline analysis

In the Results section, data are expressed and/or described as the means ± standard error (S.E.) or standard deviation (S.D.) of *N* observations, where *N* represents the number of independent experiments. Data are plotted in figures using a box and whisker plot with all points showing the minimum, 25% percentile, median, 75% percentile, and maximum values. One-way analysis of variance (ANOVA) with Tukey’s post hoc test was used to determine the parametric statistical significance. Non-parametric statistical significance was determined using the Kruskal–Wallis or Friedman test and with Dunn’s multiple comparison test or the Mann–Whitney test. Statistical significance was set at *P* < 0.05. Statistical analyses were performed using GraphPad Prism 7.0 (GraphPad Software, La Jolla, CA, USA).

## Results

### Adenylyl cyclase activator, forskolin (FSK), increases intracellular cAMP levels in human odontoblasts

**Fig. 1:**
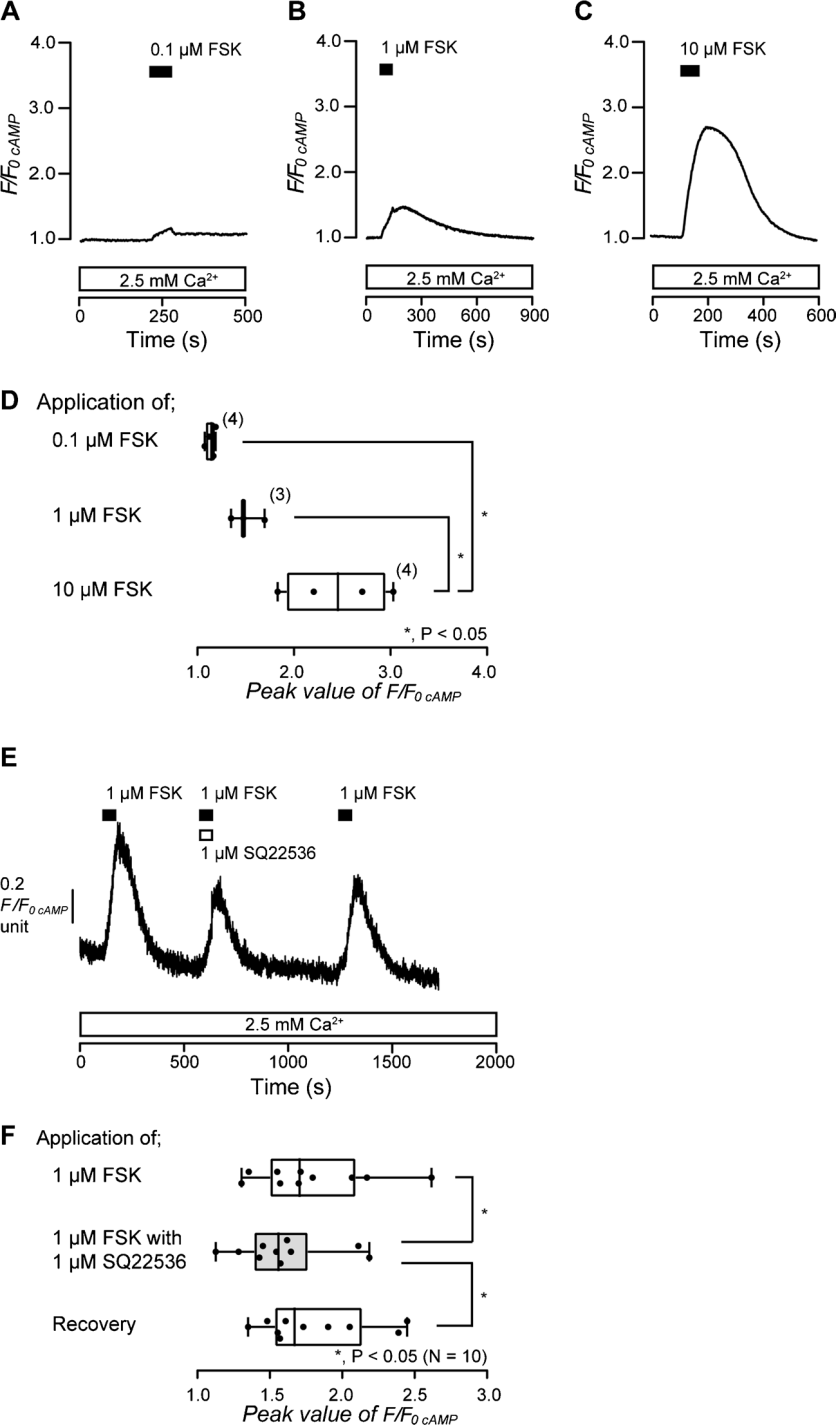
Forskolin-induced increase in intracellular cAMP level. **A** to **C** Representative traces of increase in intracellular cAMP level in response to the application of forskolin (FSK) (**A** 0.1 µM, **B** 1 µM, **C** 10 µM) (black boxes at top) in the presence of extracellular Ca^2+^ (white boxes at bottom). **D** The box and whisker plot shows the dose-dependency of FSK-induced increase in intracellular cAMP levels. Each box and whisker represents the minimum, 25% percentile, median, 75% percentile, and maximum of the peak F/F_0 cAMP_ value of four (upper column), three (middle column), and four (lower column) independent experiments. The number of each experiment is shown in parentheses. Asterisks, *P < 0.05, indicate statistically significant differences between columns (shown by solid lines). **E** Representative trace of the inhibitory effect of SQ22536 on FSK-induced increase in intracellular cAMP levels in the presence of extracellular Ca^2+^ (white box at bottom). Black boxes show the periods when 1 µM of FSK was added to the extracellular medium. The white box at the top indicates the duration of the addition of 1 µM SQ22536 to the extracellular medium. **F **The box and whisker plot of FSK-induced increase in intracellular cAMP levels without (open columns) or with (gray column) 1 µM of SQ22536. Each box and whisker represents the minimum, 25% percentile, median, 75% percentile, and maximum of the peak F/F_0 cAMP_ value of 10 independent experiments. The asterisk, *P < 0.05, indicates a statistically significant difference between columns (shown by solid lines). Recovery (lower column) shows a reversible effect of SQ22536

To demonstrate intracellular cAMP signaling elicited by activation of adenylyl cyclase in odontoblasts, we measured intracellular cAMP level during the application of an adenylyl cyclase activator with or without an adenylyl cyclase inhibitor. In the presence of extracellular Ca^2+^, application of an adenylyl cyclase activator, FSK (0.1 µM, Fig. 1A; 1 µM, Fig. 1B; 10 µM, Fig. 1C), dose-dependently increased intracellular cAMP level in HOB cells (Fig. 1A to D). The peak values were 1.13 ± 0.03 F/F_0 cAMP_ units by 0.1 µM (*N* = 4), 1.50 ± 0.10 F/F_0 cAMP_ units by 1 µM (*N* = 3), and 2.44 ± 0.27 F/F_0 cAMP_ units by 10 µM FSK (*N* = 4) (Fig. 1D). In the presence of extracellular Ca^2+^, the application of 1 µM FSK increased intracellular cAMP level to a peak value of 1.78 ± 0.13 F/F_0 cAMP_ units (*N* = 10) in HOB cells (Fig. 1E and F). The peak F/F_0 cAMP_ was significantly suppressed by the application of an adenylyl cyclase inhibitor, SQ22536 (1 µM), to 1.59 ± 0.10 F/F_0 cAMP_ units (*N* = 10; Fig. 1E and F). The inhibitory effect of SQ22536 was reversible (Fig. 1E and F). The results indicated that activation of adenylyl cyclase increased intracellular cAMP levels in human odontoblasts.

### β_2_ receptor agonist, isoproterenol (*ISO*), increases intracellular cAMP level in human odontoblasts

**Fig. 2:**
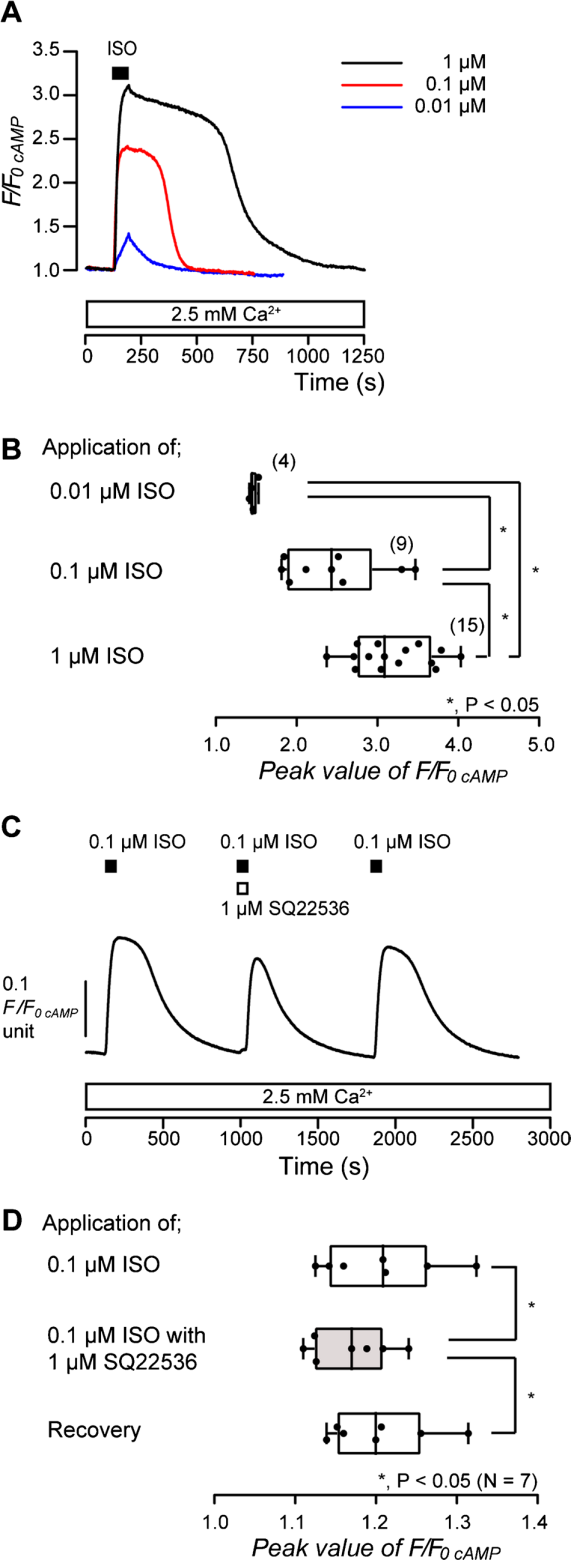
Isoproterenol-induced increase in intracellular cAMP levels. **A** Representative traces of increase in intracellular cAMP level in response to application of isoproterenol (ISO) (0.01 µM: blue line, 0.1 µM: red line, 1 µM: black line) in the presence of extracellular Ca^2+^ (white box at bottom). The period of ISO (0.01–1 µM) application is shown by a black box at the top. **B** The box and whisker plot shows the dose-dependency of ISO-induced increase in intracellular cAMP levels. Each box and whisker represents the minimum, 25% percentile, median, 75% percentile, and maximum of the peak F/F_0 cAMP_ value of four (upper column), nine (middle column), and 15 (lower column) independent experiments. The number of each experiment is shown in parentheses. Asterisks, *P < 0.05, indicate statistically significant differences between columns (shown by solid lines). **C** Representative trace of the inhibitory effect of SQ22536 on ISO-induced increase in intracellular cAMP levels in the presence of extracellular Ca.^2+^ (white box at bottom). Black boxes show periods of the addition of 0.1 µM of ISO to the extracellular medium. The white box at the top indicates the period of the addition of 1 µM of SQ22536 to the extracellular medium. **D** The box and whisker plot of ISO-induced increase in intracellular cAMP level without (open columns) or with (gray column) 1 µM SQ22536. Each box and whisker represents the minimum, 25% percentile, median, 75% percentile, and a maximum of the peak F/F_0 cAMP_ value of seven independent experiments. Asterisks, *P < 0.05, indicate statistically significant differences between columns (shown by solid lines). Recovery (lower column) shows a reversible effect of SQ22536

To demonstrate intracellular cAMP signaling in odontoblasts elicited by activation of β_2_ receptors, G_s_ protein-coupled receptors, we measured intracellular cAMP level during application of a β_2_ receptor agonist with or without adenylyl cyclase inhibitor. In the presence of extracellular Ca^2+^, application of an agonist of β_2_ receptors, ISO (0.01 µM, blue; 0.1 µM, red; 1 µM, black in Fig. 2A), increased intracellular cAMP level in a dose-dependent manner in HOB cells (Fig. 2A and B). The peak values were 1.46 ± 0.02 F/F_0 cAMP_ units upon treatment with 0.01 µM (*N* = 4), 2.44 ± 0.20 F/F_0 cAMP_ units upon treatment with 0.1 µM (*N* = 9), and 3.19 ± 0.12 F/F_0 cAMP_ units upon treatment with 1 µM ISO (*N* = 15) (Fig. 2B). In the presence of extracellular Ca^2+^, the application of 0.1 µM ISO also increased intracellular cAMP level to the peak value of 1.20 ± 0.03 F/F_0 cAMP_ units (*N* = 7) in HOB cells (Fig. 2C and D). The peak F/F_0 cAMP_ was significantly suppressed by the application of an adenylyl cyclase inhibitor, SQ22536 (1 µM), to 1.17 ± 0.02 F/F_0 cAMP_ units (*N* = 7; Fig. 2C and D). The inhibitory effect of SQ22536 was reversible (Fig. 2C and D). The results suggested that β_2_ receptor activation increased intracellular cAMP levels by stimulating adenylyl cyclase in human odontoblasts.

### Adenylyl cyclase activation induces Ca^2+^ influx in human odontoblasts

**Fig. 3:**
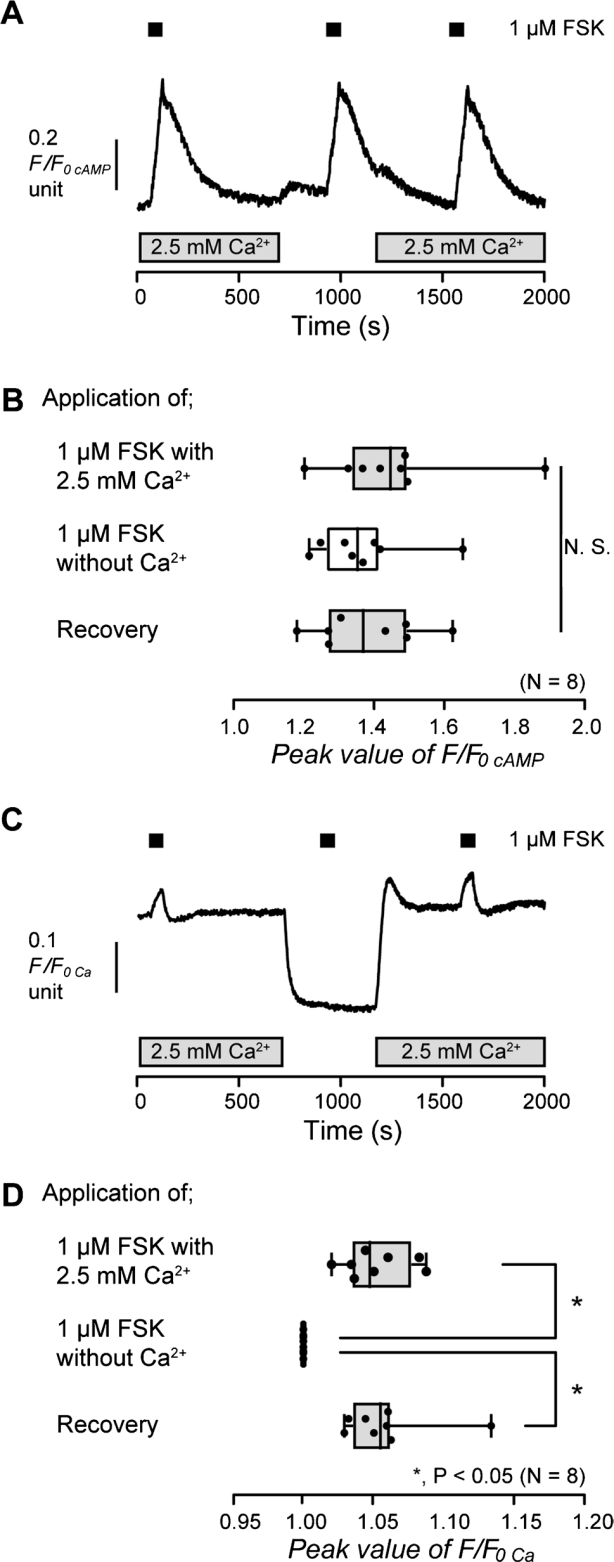
FSK-induced increase in intracellular cAMP level and [Ca^2+^]_i_ in the presence or absence of extracellular Ca^2+^. **A** and **C** Representative traces of 1 µM FSK-induced increase in intracellular cAMP levels (A) or [Ca^2+^]_i_ (C) in the presence (gray boxes at the bottom) or absence of extracellular Ca^2+^. The durations of the application of 1 µM FSK are shown by black boxes at the top. **B** and **D** The box and whisker plots of FSK-induced increase in intracellular cAMP levels (**B**) or [Ca^2+^]_i_ (**D**) with (gray columns) or without (open columns) extracellular 2.5 mM Ca^2+^. Each box and whisker represents the minimum, 25% percentile, median, 75% percentile, and maximum of the peak F/F_0 cAMP_ or F/F_0 Ca_ value of eight independent experiments. Asterisks, *P < 0.05, indicate statistically significant differences between columns (shown by solid lines). No significance between columns is denoted by N.S

There are reports that intracellular cAMP mediates intracellular Ca^2+^ signaling in various cells [9, 27, 39]. To reveal the intracellular Ca^2+^ signaling pathway induced by an increase in intracellular cAMP in odontoblasts, we investigated the alternation of intracellular cAMP level and [Ca^2+^]_i_ by application of FSK in HOB cells. In the presence of extracellular Ca^2+^, the application of 1 µM FSK increased intracellular cAMP level to the peak value of 1.46 ± 0.07 F/F_0 cAMP_ units (*N* = 8) in HOB cells (Fig. 3A and B). In the absence of extracellular Ca^2+^, the application of 1 µM FSK also increased intracellular cAMP levels (Fig. 3A). When we restored 2.5 mM Ca^2+^ to the extracellular solution, an FSK-induced increase in intracellular cAMP level was then recorded to the peak value of 1.38 ± 0.05 F/F_0 cAMP_ units (*N* = 8) (Fig. 3A and B). There were no differences in the amplitudes of the increase in intracellular cAMP levels in the presence or absence of extracellular Ca^2+^ (Fig. 3B), indicating that extracellular Ca^2+^ has no effect on the increase in intracellular cAMP level by adenylyl cyclase activation in human odontoblasts. For [Ca^2+^]_i_ measurements, in the presence of extracellular Ca^2+^, the application of 1 µM FSK increased [Ca^2+^]_i_ to the peak value of 1.05 ± 0.01 F/F_0 Ca_ units (*N* = 8) in HOB cells (Fig. 3C and D). In the absence of extracellular Ca^2+^, no FSK-induced [Ca^2+^]_i_ increase could be observed (Fig. 3C and D). The results suggested that the increase in intracellular cAMP by adenylyl cyclase activation elicited Ca^2+^ influx from the extracellular medium in odontoblasts. After removing extracellular Ca^2+^ that caused store depletion in HOB cells, the addition of extracellular Ca^2+^ (2.5 mM) induced transient [Ca^2+^]_i_ increase (Fig. 3C), showing store-operated Ca^2+^ entry (SOCE) via calcium release-activated Ca^2+^ (CRAC) channels, and/or store-operated Ca^2+^ (SOC) channels [14, 19, 46]. After [Ca^2+^]_i_ returned to the near-resting levels, the application of 1 μM FSK increased [Ca^2+^]_i_ (Fig. 3C and D).

### β_2_ receptor activation elicits Ca^2+^ influx in human odontoblasts

**Fig. 4:**
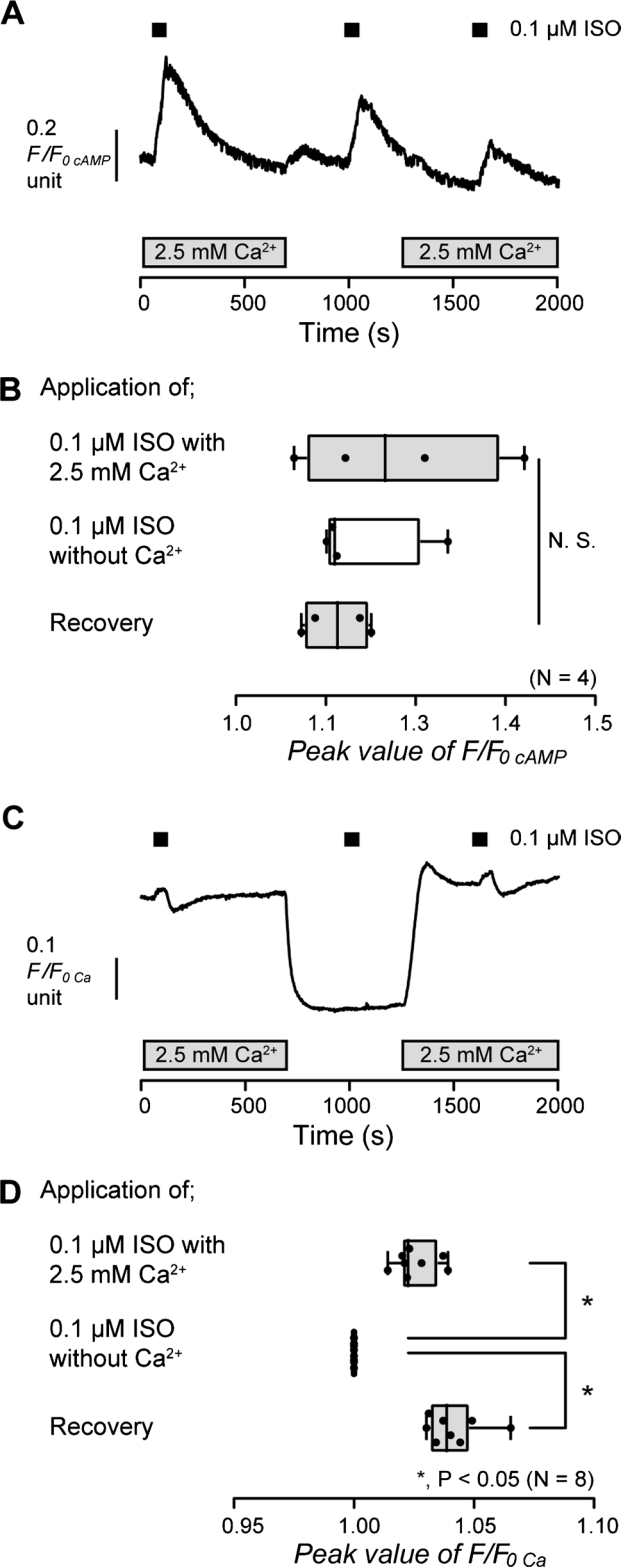
ISO-induced increase in intracellular cAMP levels and [Ca^2+^]_i_ in the presence or absence of extracellular Ca^2+^. **A** and **C** Representative traces of 0.1 µM ISO-induced increase in intracellular cAMP levels (**A**) or [Ca^2+^]_i_ (**C**) in the presence (gray boxes at the bottom) or absence of extracellular Ca^2+^. The durations of the application of 0.1 µM ISO are shown by black boxes at the top. **B** and **D** The box and whisker plots of ISO-induced increase in intracellular cAMP levels (**B**) or [Ca^2+^]_i_ (**D**) with (gray columns) or without (open columns) extracellular 2.5 mM Ca^2+^. Each box and whisker represents the minimum, 25% percentile, median, 75% percentile, and maximum of the peak F/F_0 cAMP_ or F/F_0 Ca_ value of four (**B**) and eight (**D**) independent experiments. Asterisks, *P < 0.05, indicate statistically significant differences between columns (shown by solid lines). No significance between columns is denoted by N.S

To reveal the intracellular Ca^2+^ signaling that is induced by an increase in intracellular cAMP via β_2_ receptor activation in odontoblasts, we investigated the alternation of intracellular cAMP level and [Ca^2+^]_i_ in HOB cells via the application of ISO. In the presence of extracellular Ca^2+^, the application of 0.1 µM ISO increased intracellular cAMP levels to the peak value of 1.23 ± 0.07 F/F_0 cAMP_ units (*N* = 4) in HOB cells (Fig. 4A and B). In the absence of extracellular Ca^2+^, the application of 0.1 µM ISO also increased intracellular cAMP levels (Fig. 4A). When we restored 2.5 mM Ca^2+^ in the extracellular solution, an ISO-induced increase in intracellular cAMP level was then recorded to the peak value of 1.14 ± 0.02 F/F_0 cAMP_ units (*N* = 4) (Fig. 4A and B). There were no differences in the amplitudes of the increase in intracellular cAMP levels in the presence or absence of extracellular Ca^2+^ (Fig. 4B). The results indicated that extracellular Ca^2+^ has no effect on the increase in intracellular cAMP level by β_2_ receptor activation in human odontoblasts. For [Ca^2+^]_i_ measurements, in the presence of extracellular Ca^2+^, the application of 0.1 µM ISO increased [Ca^2+^]_i_ to the peak value of 1.03 ± 0.003 F/F_0 Ca_ units (*N* = 8) in HOB cells (Fig. 4C and D). In the absence of extracellular Ca^2+^, no ISO-induced [Ca^2+^]_i_ increase could be observed (Fig. 4C and D). The results suggested that the increase in intracellular cAMP by β_2_ receptor activation is capable of activating Ca^2+^ influx from the extracellular medium in human odontoblasts. Following Ca^2+^ store depletion in HOB cells by removing extracellular Ca^2+^, the application of extracellular Ca^2+^ (2.5 mM) resulted in activation of SOCE (Fig. 4C). After [Ca^2+^]_i_ returned to the near-resting levels, the application of 0.1 µM ISO increased [Ca^2+^]_i_ (Fig. 4C and D).

### Adenylyl cyclase activation-induced Ca^2+^ influx is suppressed by a protein kinase A inhibitor

**Fig. 5:**
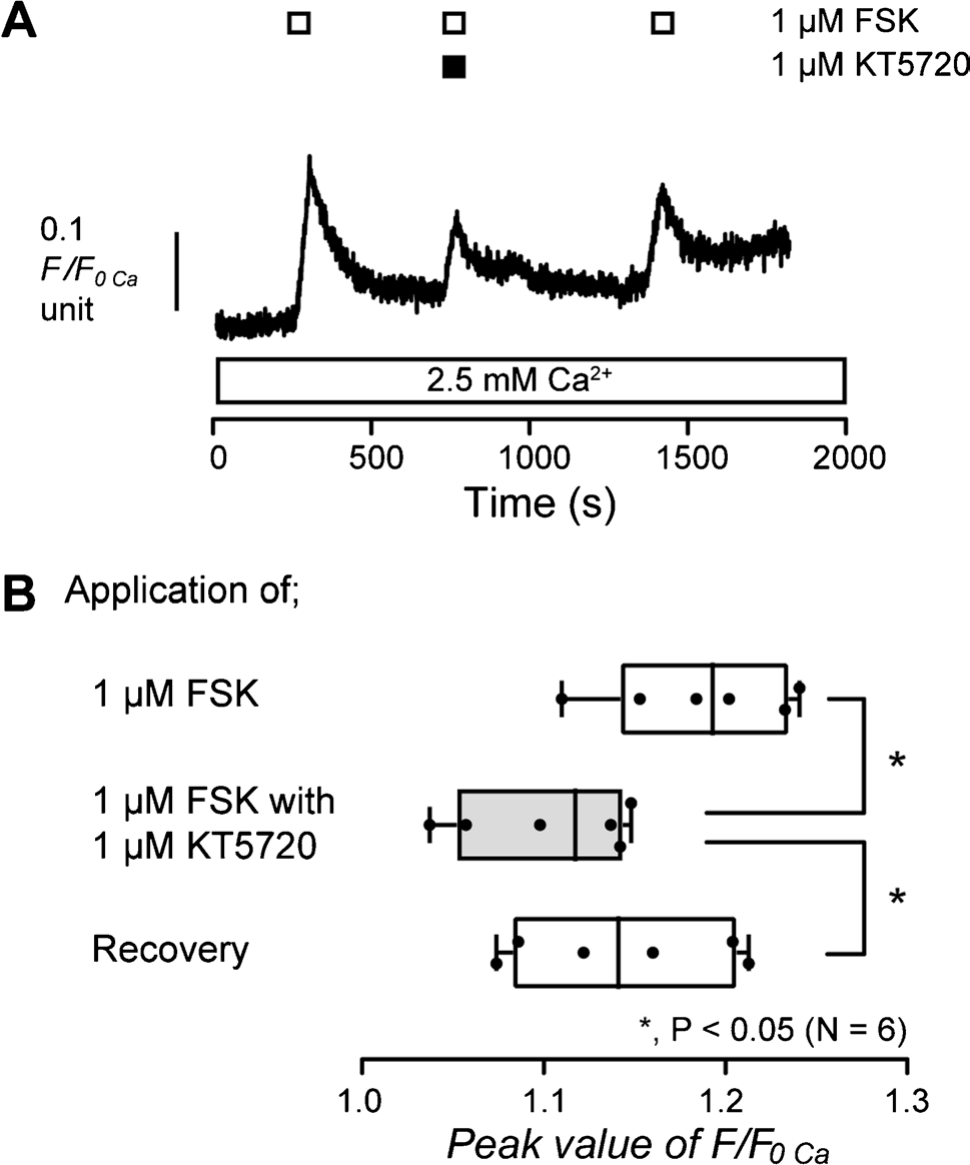
Protein kinase A inhibitor inhibited FSK-induced increase in [Ca^2+^]_i_. **A** Representative trace of the effect of a protein kinase A inhibitor on 1 µM FSK-induced increase in [Ca^2+^]_i_ in the presence of extracellular Ca^2+^ (white box at the bottom). The periods of the application of 1 µM FSK are shown by white boxes at the top. The period of the application of 1 µM KT5720 is shown by the black box at the top. **B** The box and whisker plot of FSK-induced increase in [Ca^2+^]_i_ with (gray column) or without (open columns) 1 µM of KT5720. Each box and whisker represents the minimum, 25% percentile, median, 75% percentile, and a maximum of the peak F/F_0 Ca_ value of six independent experiments. Asterisks, *P < 0.05, indicate statistically significant differences between columns (shown by solid lines). Recovery (lower column) shows a reversible effect of KT5720

Intracellular cAMP activates the PKA [28, 50]. PKA is activated by the binding of cAMP to its binding site and phosphorylates various intracellular target proteins [60]. To reveal the involvement of intracellular PKA activity in the increase in [Ca^2+^]_i_ induced by activation of adenylyl cyclase, we examined the effect of a PKA inhibitor on FSK-induced [Ca^2+^]_i_ increase. In the presence of extracellular Ca^2+^, the application of 1 µM FSK increased [Ca^2+^]_i_ to the peak value of 1.19 ± 0.02 F/F_0 Ca_ units (*N* = 6) in HOB cells (Fig. 5A and B). The increase was significantly suppressed by the application of 1 µM of PKA inhibitor, KT5720, to the peak value of 1.10 ± 0.02 F/F_0 Ca_ units (*N* = 6; Fig. 5A and B). The result showed that cAMP-mediated Ca^2+^ influx was elicited by PKA activation in human odontoblasts.

### Adenylyl cyclase activation-induced Ca^2+^ influx is sensitive to extracellular Gd^3+^, 2APB, and Zn^2+^

**Fig. 6:**
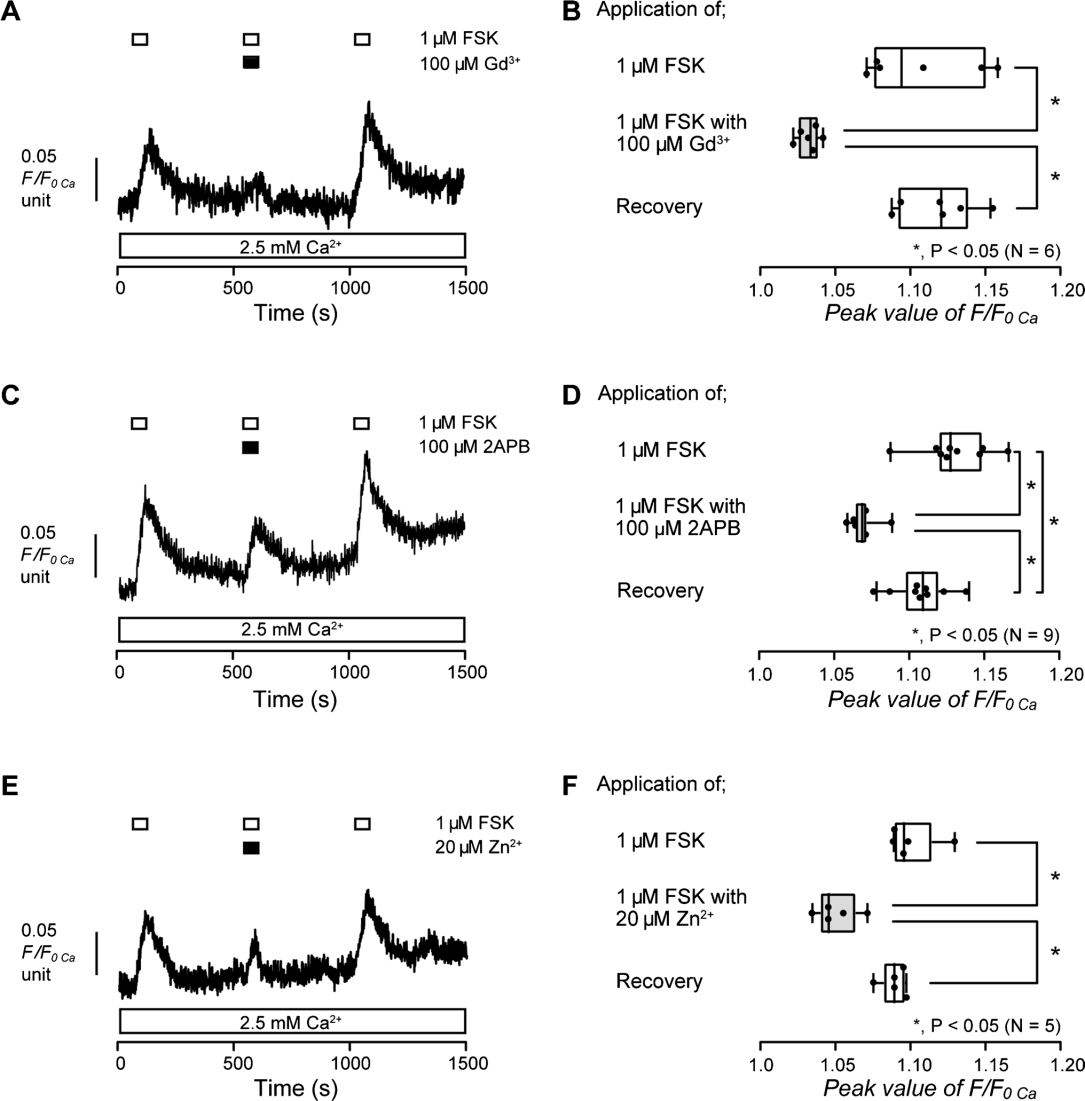
Gd^3+^, 2APB, and Zn^2+^ inhibited FSK-induced increase in [Ca^2+^]_i_. **A**, **C**, and **E** Representative traces of [Ca^2+^]_i_ increase in response to 1 µM FSK with or without 100 µM Gd^3+^ (**A**), 100 µM 2APB (**C**), or 20 µM Zn^2+^ (**E**) in the presence of extracellular Ca^2+^ (white boxes at the bottom). The durations of the application of 1 µM FSK are shown by white boxes at the top. Black boxes at the top indicate the periods of the addition of Gd^3+^ (**A**), 2APB (**C**), or Zn^2+^ (**E**) to the external solution. **B**, **D**, and **F** The box and whisker plots of FSK-induced increase in [Ca^2+^]_i_ with (gray columns) or without (open columns) 100 µM Gd^3+^ (**B**), 100 µM 2APB (**D**), or 20 µM Zn^2+^ (**F**). Peak values of FSK-induced increase in [Ca^2+^]_i_ were 1.11 ± 0.016 F/F_0 Ca_ units without Gd^3+^ (mean ± standard error (S.E.), *N* = 6) (**B**), 1.13 ± 0.008 F/F_0 Ca_ units without 2APB (mean ± S.E., *N* = 9) (**D**), and 1.10 ± 0.008 F/F_0 Ca_ units without Zn^2+^ (mean ± S.E., *N* = 5) (**F**). Peak values of FSK-induced increase in [Ca^2+^]_i_ were 1.03 ± 0.003 F/F_0 Ca_ units with 100 µM Gd^3+^ (mean ± S.E., *N* = 6) (**B**), 1.07 ± 0.003 F/F_0 Ca_ units with 100 µM 2APB (mean ± S.E., *N* = 9) (**D**), and 1.05 ± 0.006 F/F_0 Ca_ units with 20 µM Zn^2+^ (mean ± S.E., *N* = 5) (**F**). Each box and whisker represents the minimum, 25% percentile, median, 75% percentile, and maximum of the peak F/F_0 Ca_ value of six (**B**), nine (**D**), or five (**F**) independent experiments. Asterisks, *P < 0.05, indicate statistically significant differences between columns (shown by solid lines). Recovery (lower columns) shows a reversible effect of Gd^3+^ (**B**), 2APB (**D**), or Zn.^2+^ (**F**)

To reveal the pathway of [Ca^2+^]_i_ increase evoked by the activation of adenylyl cyclase, we examined the effect of non-selective Ca^2+^ channel antagonists, Gd^3+^, 2APB, and Zn^2+^ on FSK-induced increase in [Ca^2+^]_i_. In the presence of extracellular Ca^2+^, the application of 1 µM FSK increased [Ca^2+^]_i_ in HOB cells (Fig. 6A, C, and E). The FSK-induced increase in [Ca^2+^]_i_ was significantly suppressed by the application of 100 µM Gd^3+^ (Fig. 6A and B), 100 µM 2APB (Fig. 6C and D), or 20 µM Zn^2+^(Fig. 6E and F). The results suggest that activation of adenylyl cyclase evokes Ca^2+^ influx via Gd^3+^-, 2APB-, and Zn^2+^-sensitive Ca^2+^ channels in human odontoblasts.

### Adenylyl cyclase activation-induced Ca^2+^ influx is not carried by activation of L- and T-type voltage-gated Ca^2+^ channels as well as La^3+^-sensitive TRP channels in human odontoblasts

**Fig. 7:**
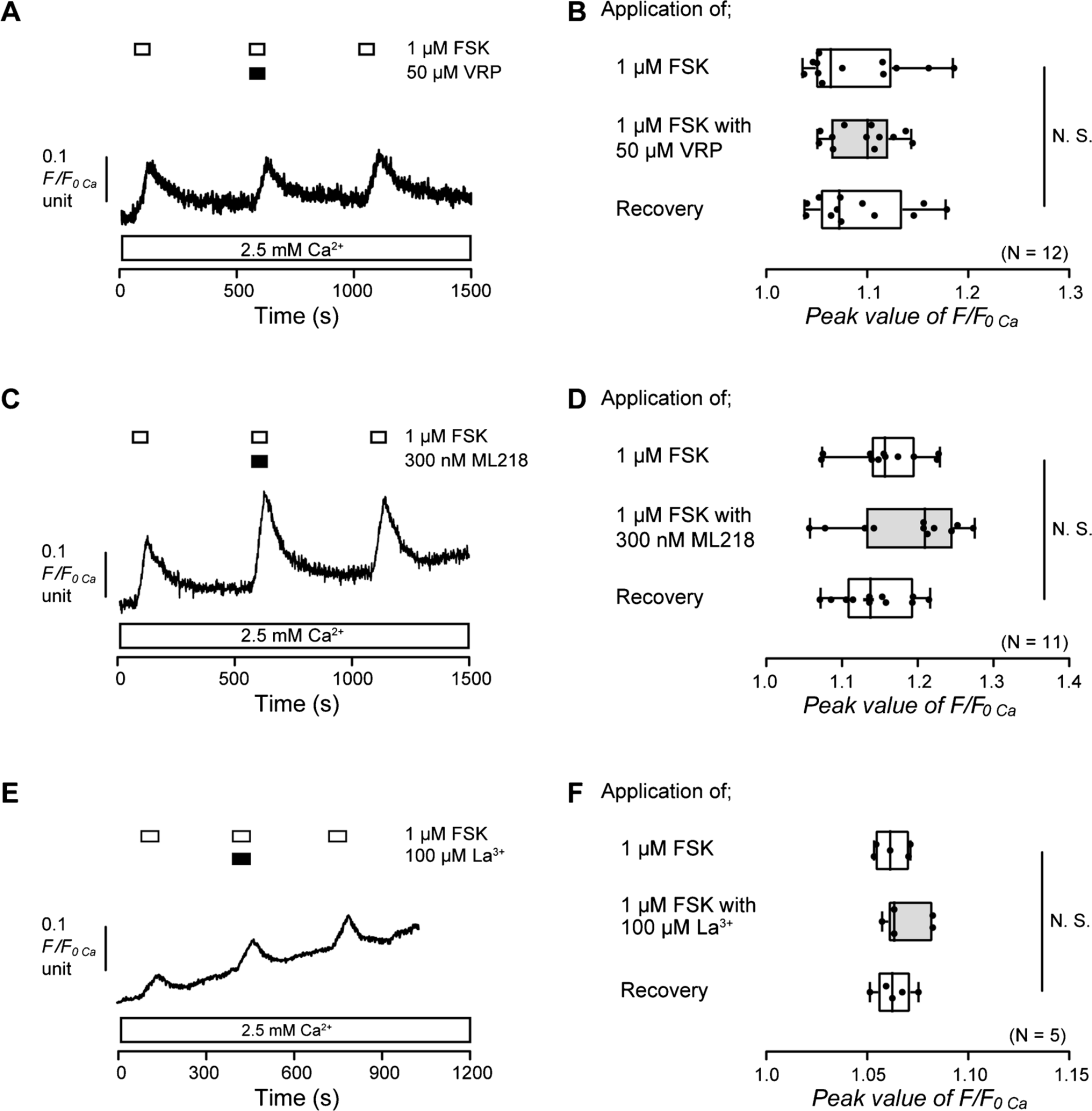
Verapamil, ML218, and La^3+^ did not affect FSK-induced increase in [Ca^2+^]_i_. **A**, **C**, and **E** Representative traces of [Ca^2+^]_i_ increase in response to 1 µM FSK with or without 50 µM verapamil (VRP) (**A**), 300 nM ML218 (**C**), or 100 µM La^3+^ (**E**) in the presence of extracellular Ca^2+^ (white boxes at the bottom). The periods of the addition of 1 µM FSK to the extracellular medium are shown by white boxes at the top. Black boxes at the top indicate periods of VRP (**A**), ML218 (**C**), or La^3+^ (**E**) addition to the external solution. **B**, **D**, and **F** Box and whisker plots of FSK-induced increase in [Ca^2+^]_i_ with (gray columns) or without (open columns) 50 µM VRP (**B**), 300 nM ML218 (**D**), or 100 µM La^3+^ (**F**). The peak values of FSK-induced increase in [Ca^2+^]_i_ were 1.09 ± 0.01 F/F_0 Ca_ units without VRP (mean ± S.E., *N* = 12) (**B**), 1.16 ± 0.02 F/F_0 Ca_ units without ML218 (mean ± S.E., *N* = 11) (**D**), and 1.06 ± 0.004 F/F_0 Ca_ units without La^3+^ (mean ± S.E., *N* = 5) (**F**). The peak values of FSK-induced increase in [Ca^2+^]_i_ were 1.09 ± 0.01 F/F_0 Ca_ units with VRP (mean ± S.E., *N* = 12) (**B**), 1.18 ± 0.02 F/F_0 Ca_ units with ML218 (mean ± S.E., *N* = 11) (**D**) and 1.07 ± 0.005 F/F_0 Ca_ units with La^3+^ (mean ± S.E., *N* = 5) (**F**). Each box and whisker represents the minimum, 25% percentile, median, 75% percentile, and a maximum of the peak F/F_0 Ca_ value of 12 (**B**), 11 (**D**), or 5 (**F**) independent experiments. No significance between columns is denoted by N.S

Gd^3+^ is known to inhibit L- and T-type voltage-gated Ca^2+^ channels (VGCC) as well as some TRP channels [25, 26]. Intracellular cAMP is also recognized to activate Ca^2+^ influx via L- and T-type VGCC [9, 27, 39]. Hence, we investigated the participation of L- and T-type VGCC in the FSK-induced increase in [Ca^2+^]_i_ in HOB cells. In the presence of extracellular Ca^2+^, the application of 1 µM FSK increased [Ca^2+^]_i_ in HOB cells (Fig. 7A and C). The [Ca^2+^]_i_ increases were not inhibited by the application of 50 µM of an L-type VGCC blocker, VRP (Fig. 7A and B), or 300 nM of a T-type VGCC blocker, ML218 (Fig. 7C and D). La^3+^ is also known to suppress some TRP channels as well as L- and T-type VGCC [33, 38]. In the presence of extracellular Ca^2+^, the application of 1 µM FSK increased [Ca^2+^]_i_ in HOB cells (Fig. 7E). The [Ca^2+^]_i_ increases were not inhibited by the application of 100 µM La^3+^ (Fig. 7E and F). These results suggested that cAMP-mediated Ca^2+^ influx was not carried by L- and T-type VGCC, as well as La^3+^-sensitive TRP channels in human odontoblasts.

### Human odontoblasts express calcium homeostasis modulator 1 (CALHM1) as Ca^2+^ entry pathway

**Fig. 8:**
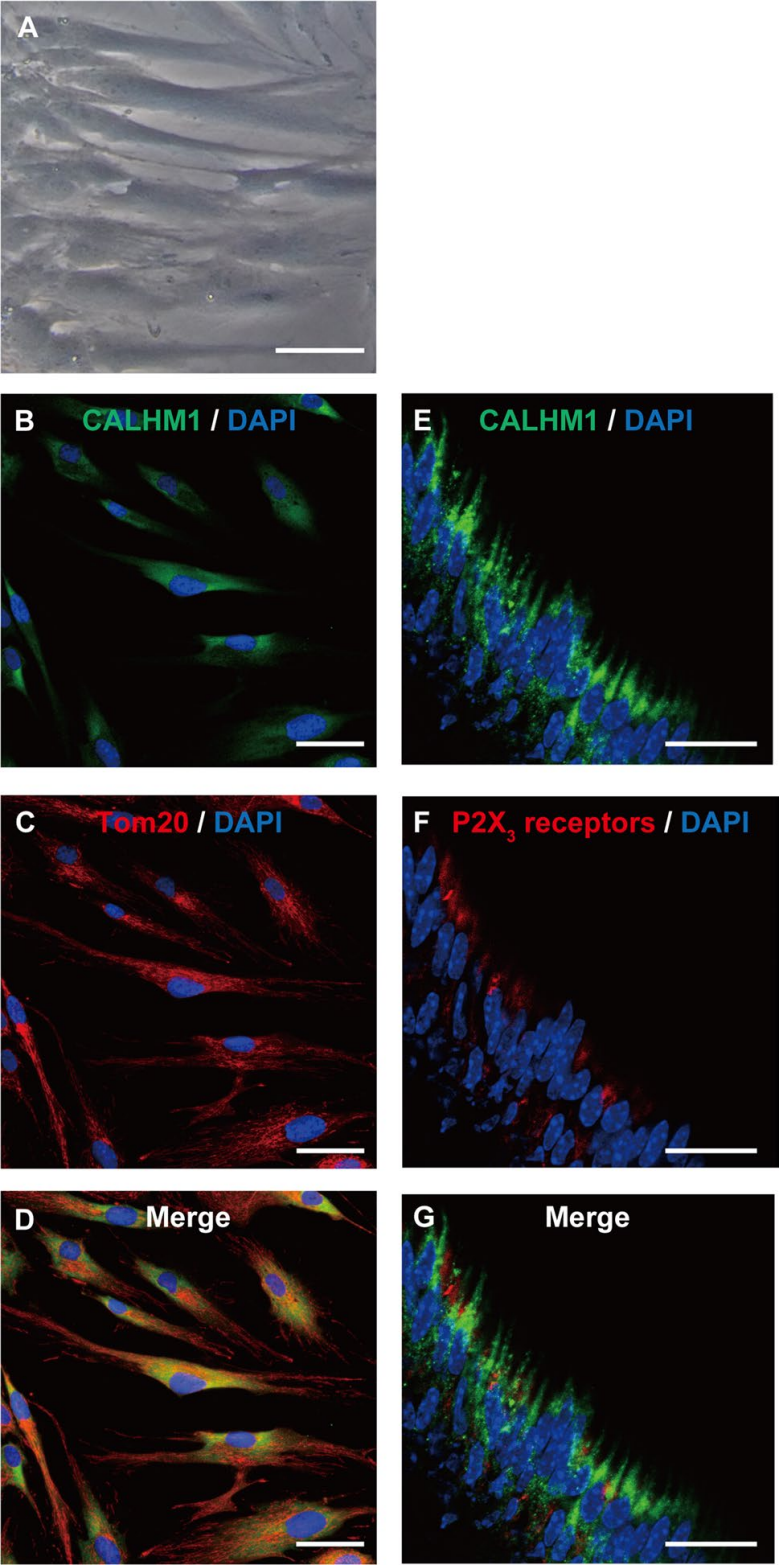
Immunofluorescence analysis of CALHM1 and Tom20 in HOB cells and of CALHM1 and P2X_3_ receptors in mouse mandibular molar dental pulp. **A** Observation of HOB cells in phase contrast. **B** and **C** HOB cells were positive for CALHM1 (green in **B**) and Tom20 (red in **C**). **D** Triple immunofluorescence staining with antibodies against the CALHM1 (green) and Tom20 (red). The nuclei are shown in blue. Scale bars, 50 µm. **E** to **G** Tall columnar cells localized on the dental pulp periphery were positive for CALHM1 (green in **E** and **G**). Immunoreactivity was also observed for P2X_3_ receptors (red in **F** and **H**). The nuclei are shown in blue. Scale bars, 20 µm

CALHM1 is a well-known Ca^2+^ permeable channel that is sensitive to extracellular Gd^3+^, 2APB, and Zn^2+^ but not to La^3+^. CALHM1 also acts as an ATP-releasing channel. To determine whether odontoblasts express CALHM1, we examined the protein expression of CALHM1 and Tom20 in HOB cells using immunofluorescence staining. Tom20 is a mitochondrial outer membrane marker. Phase-contrast imaging of HOB cells cultured in basal medium (Fig. 8A) revealed a columnar-like spindle shape. Immunofluorescence analysis demonstrated that the HOB cells were independently positive for CALHM1 (green; Fig. 8B and D) and Tom20 (red; Fig. 8C and D) expression. We further examined the localization patterns of CALHM1 and P2X_3_ receptors in the dental pulp of mouse mandibular molars. In a previous study, no functional expression of P2X_3_ receptors was found in odontoblasts [37, 48]; however, P2X_3_ receptor expression was found on neurofilament heavy chain (NF-H)-positive A neurons [37]. In line with previous results, we found immunoreactivity of P2X_3_ receptors (red; Fig. 8F and G) close to CALHM1 positive staining in the peripheral region of the dental pulp (green; Fig. 8E and G).

### CALHM1 knockdown by shRNA in human odontoblasts suppresses adenylyl cyclase activation-induced Ca^2+^ influx

**Fig. 9:**
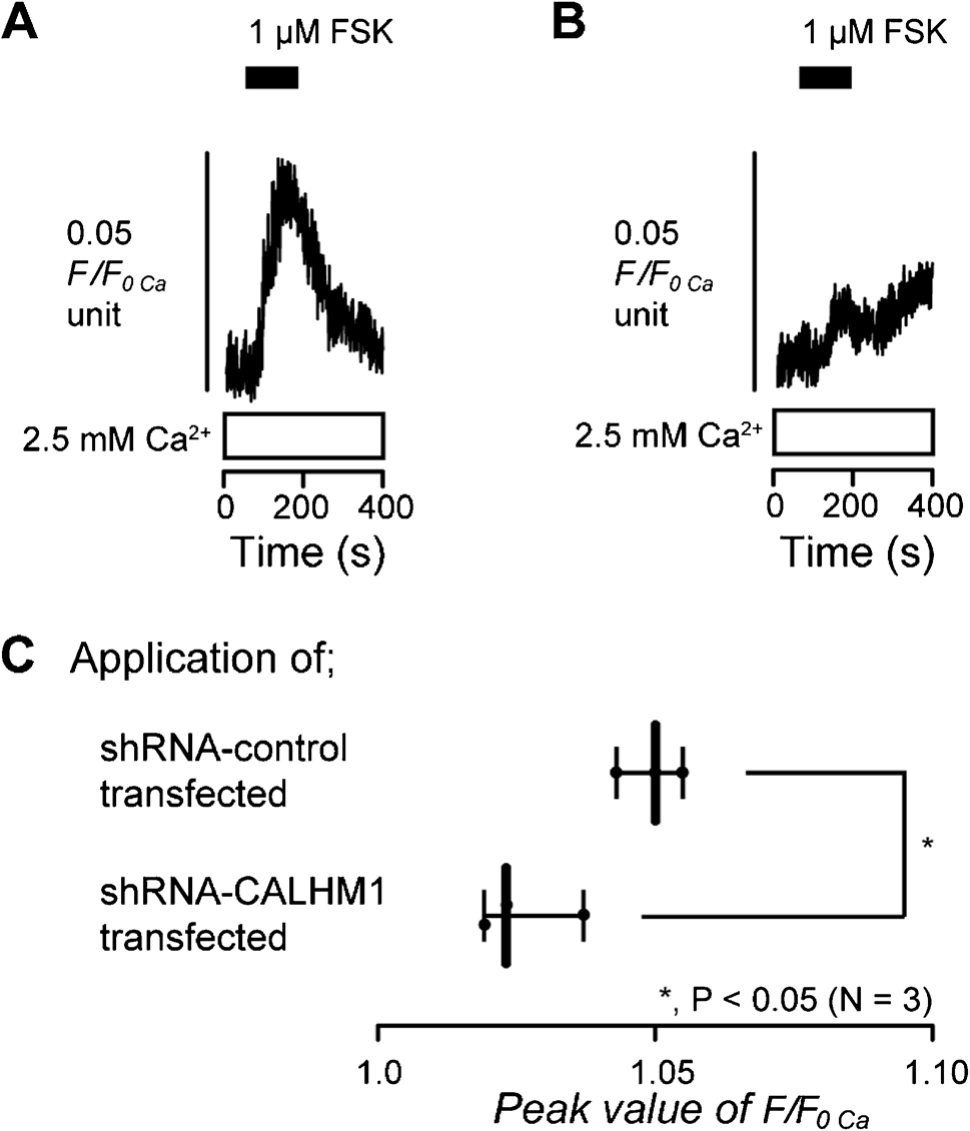
CALHM1 knockdown by shRNA. **A** and **B** Representative traces of [Ca^2+^]_i_ increases in response to 1 µM FSK in the presence of extracellular Ca^2+^ (white boxes at the bottom) in HOB cells transfected by shRNA including an empty vector control (shRNA-control transfected) (**A**) and a vector specific for human CALHM1 (shRNA-CALHM1 transfected) (**B**). The durations of the addition of 1 µM FSK to the extracellular medium are shown by black boxes at the top. **C** Box and whisker plot represents the peak value of FSK-induced increase in [Ca^2+^]_i_ in HOB cells transfected by shRNA including an empty vector control (upper column) and a vector specific for human CALHM1 (lower column). Each box and whisker represents the minimum, 25% percentile, median, 75% percentile, and a maximum of the peak F/F_0 Ca_ value of three independent experiments

To determine the participation of CALHM1 in the increased [Ca^2+^]_i_ induced by the activation of adenylyl cyclase, we investigated the FSK-induced increase in [Ca^2+^]_i_ using HOB cells transfected with shRNA targeting human CALHM1 (shRNA-CALHM1 transfected HOB cells) or with an empty vector control (shRNA-control transfected HOB cells). In the presence of extracellular Ca^2+^, the application of 1 µM FSK increased [Ca^2+^]_i_ to the peak value of 1.05 ± 0.003 F/F_0 Ca_ units (*N* = 3) in shRNA-control transfected HOB cells (Fig. 9A and C). The [Ca^2+^]_i_ increase was significantly inhibited in shRNA-CALHM1 transfected HOB cells, compared to that observed in the shRNA-control cells, showing a peak value of 1.03 ± 0.005 F/F_0 Ca_ units (*N* = 3) (Fig. 9B and C). The results suggest that the Ca^2+^ influx induced by the activation of adenylyl cyclase is mediated by CALHM1 in human odontoblasts.

### Adenylyl cyclase activation negatively modulates mineralization level by human odontoblasts

**Fig. 10:**
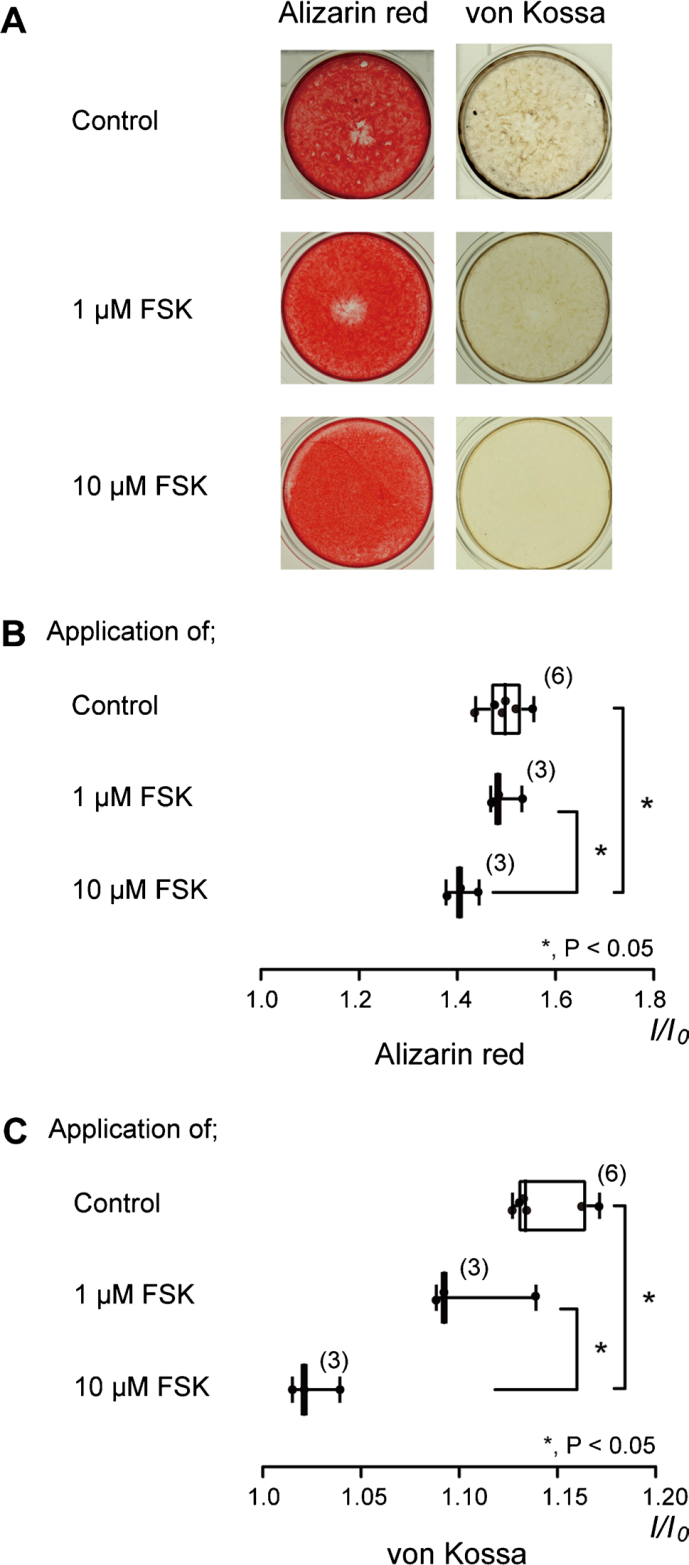
FSK decreased mineralization level by HOB cells. **A** We cultured HOB cells for 21 days in a mineralization medium without FSK (upper columns) or with FSK (1 µM: middle columns, 10 µM: lower columns) at pH 7.4. The left columns show alizarin red staining, and the right columns show von Kossa staining. **B** and **C** Mineralization levels without FSK (open columns) and with 1 µM (middle gray columns) or 10 µM FSK (lower gray columns) were assessed by alizarin red (**B**) and von Kossa staining (**C**). The mineralization levels observed via alizarin red staining were 1.50 ± 0.04 I/I_0_ units in the absence of FSK (mean ± standard deviation (S.D.), *N* = 6) (as controls), 1.49 ± 0.03 I/I_0_ units in the presence of 1 µM FSK (mean ± S.D., *N* = 3), and 1.41 ± 0.03 I/I_0_ units in the presence of 10 µM FSK (mean ± S.D., *N* = 3) (**B**). The mineralization levels observed via von Kossa staining were 1.14 ± 0.02 I/I_0_ units in the absence of FSK (mean ± S.D., *N* = 6) (as controls), 1.11 ± 0.03 I/I_0_ units in the presence of 1 µM FSK (mean ± S.D., *N* = 3), and 1.03 ± 0.01 I/I_0_ units in the presence of 10 μM FSK (mean ± S.D., *N* = 3) (**C**). Each box and whisker in **B** and **C** represent the minimum, 25% percentile, median, 75% percentile, and maximum mineralization levels from six (upper columns), three (middle columns), and three (lower columns) independent experiments. The number of each experiment is shown in parenthesis. Statistically significant differences between columns (shown by solid lines) are denoted by asterisks. * *P* < 0.05

We examined the effect of FSK on the mineralization capacity by HOB, to demonstrate participation of the increase in intracellular cAMP level in mineralization. We compared mineralization levels after culture for 21 days in a mineralization medium containing 1.8 mM Ca^2+^ between the cells subjected with or without FSK (1 or 10 µM). The mineralization level was assessed using alizarin red (left columns) and von Kossa (right columns) staining (Fig. 10A). Mineralization levels by HOB cells cultured in the mineralization medium without FSK were used as a control, as shown in Fig. 10. In both the alizarin red and von Kossa stainings, adding 1 µM FSK to the mineralization medium had no effect on the mineralization level, whereas the addition of 10 µM FSK to the medium significantly decreased the mineralization level (Fig. 10A to C). The results indicated that adenylyl cyclase activation suppressed mineralization levels by human odontoblasts.

## Discussion

In the present study, we demonstrated that activation of adenylyl cyclase and β_2_ receptors elicited an increase in intracellular cAMP. Subsequently, intracellular cAMP signaling activated Ca^2+^ signaling through the Gd^3+^-, 2APB-, and Zn^2+^-sensitive Ca^2+^ channel, CALHM1, via PKA in odontoblasts.

We investigated the Ca^2+^ permeable ion channels activated by intracellular cAMP following adenylyl cyclase activation in odontoblasts. FSK-induced Ca^2+^ influx was found to be suppressed by Gd^3+^, an antagonist of non-selective Ca^2+^ channels, including mechanosensitive ion channels, VGCC, and several types of TRP channels [3, 25, 26, 32, 56]. The influx was also inhibited by 2APB, a broad-spectrum TRP channel antagonist [2, 4, 58]. These results imply that Gd^3+^- and 2APB-sensitive intracellular cAMP-induced Ca^2+^ influx is mediated by mechanosensitive Piezo channels as well as TRP canonical subfamily member 1 (TRPC1), TRPC6, and TRP melastatin subfamily member 7 (TRPM7) channels. As we did not treat the odontoblasts with mechanical stimulation or pharmacological modulators of the Piezo channels, the contribution of these channels to cAMP-activated Ca^2+^ influx is highly unlikely. Odontoblasts are known to express the TRPC1 [49], TRPC6 [59], and TRPM7 [35, 57] channels; notably, the TRPC1 [40], TRPC6 [5, 15], and TRPM7 [16, 34] channels are known to be sensitive to La^3+^. However, in the present study, FSK-induced Ca^2+^ influx was not suppressed by La^3+^, indicating that Ca^2+^ influx induced by increased intracellular cAMP was not mediated by the TRPC1, TRPC6, and TRPM7 channels. Increased intracellular cAMP levels have been described to elicit Ca^2+^ influx via the T-type VGCC in porcine olfactory receptor neurons [9]. An increase in intracellular cAMP also activates L-type VGCC in mouse taste receptor cells and neural progenitor cells in the subventricular zone at E14.5, resulting in Ca^2+^ influx [27, 39]. Therefore, L- and T-type VGCC may be candidates for the channels of FSK-induced Ca^2+^ influx. Odontoblasts are thought to express both L- and T-type VGCC [1, 11, 17]. L-type VGCC is almost completely blocked by 200 nM Gd^3+^ [3], whereas T-type VGCC is suppressed by Gd^3+^ with a half-maximal (50%) inhibitory concentration (IC_50_) of 87 nM [25, 26]. In the present study, although a high concentration of Gd^3+^ (100 µM) inhibited L- and T-type VGCC, FSK-induced Ca^2+^ influx was not suppressed by the application of selective L- or T-type VGCC antagonists or by a nonselective VGCC antagonist La^3+^ in HOB cells. Therefore, Ca^2+^ influx induced by an increase in intracellular cAMP was not mediated by L- and T-type VGCC, TRPC1, TRPC6, or TRPM7, or by mechanosensitive ion channels in odontoblasts.

CALHM1 is a physiologically important non-selective cation channel of the plasma membrane that participates in neuronal excitability and taste perception [30], and is regulated by membrane voltage and extracellular Ca^2+^ concentration [30, 31]. CALHM1 is permeable to cations and ATP [51]. Recently, CALHM1 in type II taste receptor cells was shown to act as a transmitter-releasing pathway of ATP and to establish intercellular communication between type II taste receptor cells and gustatory afferents [52]. Action potentials evoke ATP release from CALHM1 in type II taste receptor cells, and the released ATP binds to P2X/P2X_3_ receptors in primary gustatory afferents [31], to produce gustation. Gd^3+^, Zn^2+^, and 2APB are well known to act as CALHM1 inhibitors [30]. FSK-induced Ca^2+^ influx was inhibited by Zn^2+^, 2APB, and Gd^3+^ in HOB cells. Additionally, FSK-induced Ca^2+^ influx was suppressed by CALHM1 knockdown in HOB cells. Therefore, our results indicate that an increase in intracellular cAMP elicited by G_s_ protein-coupled receptor activation evokes Ca^2+^ influx via CALHM1 activation in odontoblasts. Further, Ca^2+^ influx induced by an increase in intracellular cAMP was found to be sensitive to PKA inhibition, suggesting that PKA likely activates CALHM1. However, to date, there have been no reports describing PKA-mediated CALHM1 activation. Further studies are thus needed to demonstrate PKA-induced CALHM1 activation in odontoblasts.

Odontoblasts establish intercellular communication with the Aδ primary afferent of TG neurons in dental pulp via ATP to induce dentinal pain, as sensory receptor cells [37, 42, 45]. Ca^2+^ influx via mechanosensitive ion channel activation following stimulation of the dentin surface mediates ATP release from pannexin-1 channels (PANX-1) in odontoblasts. ATP acts as an intercellular transmitter and binds to P2X_3_ receptors in the Aδ-afferent in dental pulp. In this study, activation of G_s_ protein-coupled receptors induced Ca^2+^ influx via CALHM1 in odontoblasts. It is well-documented that CALHM1 activation releases ATP as an intercellular transmitter. CALHM1 in type II taste receptor cells is expressed adjacent to the mitochondria and is located close to the P2X ATP receptors/channels in the afferent nerves [52]. Using immunofluorescence analysis, we demonstrated CALHM1 expression in cells localized in the peripheral area of the dental pulp, showing a tall, columnar shape. CALHM1 immunoreactivity was also observed close to the mitochondrial outer membrane, as well as to P2X_3_ receptor-immunoreactivity. Notably, odontoblasts express P2X_4_ and P2X_7_, but not P2X_3_ receptors, whereas NF-H-positive TG neurons (A neurons) located at the periphery of the dental pulp (i.e., the odontoblast layer) express P2X_3_ receptors. Together with previous results, the present study indicated that P2X_3_ receptor on the dental pulp Aδ-afferent localizes adjacent to CALHM1 in odontoblasts. Although further studies are needed, this finding indicates that ATP released from CALHM1 by the activation of G_s_ protein-coupled receptors may modulate neurotransmission between odontoblasts and neurons for sensory transduction sequences in the development of dentinal pain via P2X_3_ receptors in TG neurons in the dental pulp [24, 45].

However, in the present study, CALHM1 inhibitors and CALHM1 knockdown by shRNA did not abolish FSK-induced Ca^2+^ influx. This suggests two possibilities: (1) CALHM1 was not completely knocked down by shRNA-CALHM1, and (2) Ca^2+^-permeable channels, except for CALHM1, participate in FSK-induced Ca^2+^ influx. Although La^3+^ did not affect FSK-induced Ca^2+^ influx, it is a nonselective Ca^2+^ channel antagonist affecting VGCC as well as the TRPC1, TRPC6, and TRPM7 channels. Thus, we cannot exclude the possibility that these channels may also contribute to the Ca^2+^ influx induced by increased intracellular cAMP in odontoblasts. Further studies are thus required to elucidate the residual FSK-activated Ca^2+^ influx pathways.

ATP released by the activation of G_s_ protein-coupled receptors through CALHM1 may bind to P2X_4_ and P2X_7_ receptors in odontoblasts, establishing intercellular odontoblast communication. In our previous study, when we applied whole-cell patch-clamp recordings on mouse odontoblast lineage cells, we observed ATP-induced inward currents composed of Na^+^ and Ca^2+^ conductance; the main ionic component of cation permeability for the P2X_4_ receptor was Ca^2+^, whereas that for P2X_7_ was Na^+^ in odontoblasts [48]. The concentration of released ATP by external dentin cold stimulation has been reported to be in the “nM” range in an in vitro human tooth perfusion model [7, 29]. The P2X receptor subtypes expressed in odontoblasts need ~ 1000 times as high a concentration of extracellular ATP for their activation (ca. 50–100 µM range), compared to the ATP concentration in dentin stimulation-induced release. Therefore, ATP released from the CALHM1 and P2X_4/7_ receptors in odontoblasts is unlikely to mediate intercellular odontoblast-odontoblast communication [36, 42].

We found that an increase in intracellular cAMP levels suppressed odontoblast mineralization. In our previous study, activation of CGRP receptors, which are G_s_ protein-coupled receptors, increased intracellular cAMP levels by stimulating adenylyl cyclase in rat odontoblasts [41]. CGRP receptor activation inhibited mineralization by odontoblasts, and this inhibitory effect was reversibly suppressed by the adenylyl cyclase inhibitor, SQ22536 [41]. Further, systemic administration of a β_2_ receptor “antagonist” was found to increase tertiary dentinogenesis in rats [10]. These reports are consistent with the results of the present study, which shows that an increase in intracellular cAMP inhibits mineralization by odontoblasts. Therefore, increased intracellular cAMP levels in odontoblasts negatively modulate dentin formation and mineralization. Previously, we reported that inhibition of Na^+^-Ca^2+^ exchange (NCX) or plasma membrane Ca^2+^-ATPase (PMCA) decreased mineralization by odontoblasts [18, 20, 44, 54]. These results indicate that NCX and PMCA are involved in mineralization. Therefore, the intracellular cAMP-dependent pathway may inhibit NCX or PMCA in odontoblasts, resulting in decreased dentin mineralization. However, further studies are required to investigate this aspect further.

Pain, including tooth pain, activates the systemic sympathetic nervous system. Sympathetic nerves in the dental pulp originate from the superior cervical ganglion and are frequently distributed in the coronal pulp [47]. These nerves innervate the blood vessels in the dental pulp of noncarious teeth and contribute to the regulation of pulpal blood flow [8, 13]. In normal dental pulp, sympathetic nerve fibers are adjacent to each odontoblast cell body, whereas sympathetic innervation exists only slightly [47]. In injured dental pulp, sympathetic nerve fibers sprout toward odontoblast cell bodies during reparative dentin formation after dental pulp exposure [12, 47]. In decayed teeth, sympathetic innervation exists in the subodontoblastic plexus of Raschkow and the odontoblastic layer [8]. Therefore, noradrenaline released from sympathetic nerve fibers may activate β_2_ receptors in odontoblasts localized on cell bodies and their processes [10], resulting in increased intracellular cAMP via stimulation of adenylyl cyclase and inhibition of reactionary dentin formation. In contrast, parasympathetic innervation of the human dental pulp is controversial [61]. Odontoblasts express muscarinic cholinergic receptors and G_q_ protein-coupled receptors [19, 46]. Assuming that parasympathetic nerves are distributed in the dental pulp, acetylcholine released from the parasympathetic nerve terminal binds to muscarinic cholinergic receptors in odontoblasts. Its receptor activation induces Ca^2+^ release from Ca^2+^ stores following the phospholipase C signaling pathway and elicits SOCE via the CRAC and/or SOC channels [19, 46]. Increased intracellular Ca^2+^ is then extruded into the extracellular medium via NCX and PMCA, resulting in dentin formation. Therefore, odontoblasts are likely innervated by the autonomic nervous system, and their cellular functions may be regulated by the nervous system, which has dual innervation. Dentin formation is likely negatively mediated by sympathetic nerves but positively mediated by parasympathetic nerves. Further, we previously showed that CGRP, a ligand of CGRP receptors, is expressed in peptidergic C neurons among the TG neurons. CGRP is released from neurons during direct mechanical stimulation, which mimics the increased tissue pressure during dental pulp inflammation. Increasing the tissue pressure inside the dental pulp promotes the tightness of the C-fibers as mechanical stimulation [41]. CGRP released from stimulated TG neurons activates intracellular cAMP signaling via CGRP receptor activation in odontoblasts neighboring the stimulated TG neurons, resulting in the inhibition of mineralization by odontoblasts [41]. Thus, the adenylyl cyclase signaling cascade following G_s_ protein-coupled receptor activation appears to negatively modulate dentinogenesis. Although intracellular cAMP signaling via the activation of G_s_ protein-coupled receptors elicits Ca^2+^ influx through CALHM1 in odontoblasts, further studies are needed to clarify the role of CALHM1 in cAMP-mediated odontoblast cellular functions.

## Conclusion

We showed that the activation of adenylyl cyclase mediates an increase in intracellular cAMP levels. The increased intracellular cAMP-dependent pathway, including PKA, subsequently activates Ca^2+^ entry via CALHM1 in odontoblasts. There is considerable interest in understanding whether CALHM1 acts in the ATP-releasing pathway that participates in dentinal pain generation in odontoblasts. We also revealed the inhibitory effect of intracellular cAMP on the mineralization by odontoblasts. Therefore, we suggest that the activation of G_s_ protein-coupled receptors in odontoblasts and cAMP-PKA signaling, as well as cAMP-induced Ca^2+^ entry via CALHM1, participate in regulating cellular functions, such as dentin formation, mineralization, and generation of dentinal pain.

## Data Availability

No datasets were generated or analysed during the current study.
